# Association between *Staphylococcus aureus* colonization and clinical improvement in pediatric atopic dermatitis treated with dupilumab: a pilot study

**DOI:** 10.3389/fmed.2026.1836967

**Published:** 2026-05-18

**Authors:** Cristiana Indolfi, Angela Klain, Giulio Dinardo, Simone Colosimo, Serena Ferrara, Carolina Grella, Massimiliano Galdiero, Anna De Filippis, Valentina Fiore, Michele Miraglia del Giudice

**Affiliations:** 1 Department of Woman, Child and General and Specialized Surgery, University of Campania "Luigi Vanvitelli, Naples, Italy; 2 Department of Experimental Medicine, University of Campania "Luigi Vanvitelli", Naples, Italy; 3 Complex Operative Unit of Virology and Microbiology, University Hospital of Campania, Naples, Italy

**Keywords:** atopic dermatitis, biological therapy, dupilumab, microbiome, pediatric dermatology, quality of life, skin microbiota, *Staphylococcus aureus*

## Abstract

**Background:**

Moderate-to-severe atopic dermatitis (AD) in children is characterized by impaired skin barrier function, type 2 inflammation, and frequent *Staphylococcus aureus* colonization, contributing to disease severity and risk of superinfection. Dupilumab, an anti–IL-4/IL-13 receptor monoclonal antibody, improves clinical outcomes in pediatric AD, but longitudinal data on culture-based skin and nasal microbial changes remain limited.

**Objective:**

To assess dupilumab efficacy in children with moderate-to-severe AD unresponsive to conventional therapy and to describe skin and nasal microbial colonization patterns at the 12-month time point compared with moderate AD receiving conventional topical therapy and healthy controls.

**Methods:**

Prospective observational study. Children aged 6–16 years were enrolled in three groups: (a) moderate-to-severe AD starting dupilumab (assessments at baseline, 3, 6, and 12 months); (b) moderate AD receiving conventional topical therapy not eligible for biologic therapy; and (c) age-matched healthy controls. Outcomes included Eczema Area and Severity Index (EASI), Children’s Dermatology Life Quality Index (C-DLQI), and Peak Pruritus Numerical Rating Scale (NRS). At 12 months, nasal and skin e-Swabs were cultured; isolates were identified by MALDI-TOF with antimicrobial susceptibility testing. Longitudinal changes were analyzed using the Friedman test (*p* < 0.05).

**Results:**

Ten dupilumab-treated children (mean age 13 years; 60% males) showed rapid and sustained improvement in C-DLQI (median 13.5 to 3 at 3 months; 3.5 at 12 months), EASI (24.5 to 5.65 at 3 months, 1.2 at 12 months), and pruritus (NRS 10 to 4.5 at 3 months; 5.5 at 12 months). No adverse events or discontinuations occurred. At 12 months, nasal *S. aureus* colonization was detected in 2/10 dupilumab-treated patients versus 4/10 moderate AD receiving conventional topical therapy and 1/10 controls; skin *S. aureus* was absent in dupilumab-treated patients but present in 8/10 children with moderate atopic dermatitis receiving conventional topical therapy and 0/10 controls.

**Conclusion:**

Dupilumab provides sustained clinical benefit and, in this cross-sectional assessment of a small pilot cohort, is associated with lower *S. aureus* colonization and the presence of commensal staphylococci in pediatric atopic dermatitis.

## Highlights

- The use of dupilumab in children with moderate-to-severe atopic dermatitis is associated with marked improvement in severity scores (EASI), pruritus (NRS), and quality of life (C-DLQI) as early as 3–6 months after initiation.
- At the same time, it is associated with lower *Staphylococcus aureus* colonization and the presence of commensal skin and nasal bacteria.
- The clinical effect is maintained for 12 months and is accompanied by good tolerability, suggesting that dupilumab not only controls inflammation but may also be associated with a pattern potentially consistent with a more physiologic skin and nasal colonization profile.
- These microbiological findings, derived from a cross-sectional assessment of a small pilot cohort, should be interpreted with caution as exploratory findings.

## 1 Introduction

Atopic dermatitis (AD) is a chronic, relapsing inflammatory skin disorder characterized by intense pruritus, eczematous lesions with variable morphology and distribution depending on age, and a disrupted epidermal barrier that predisposes to xerosis, microbial colonization, and increased transepidermal water loss (1). The disease course is typically fluctuating, with acute flares and periods of partial remission, and it imposes a substantial burden on patients and families, leading to impaired sleep, psychosocial distress, and a significantly reduced quality of life (QoL) (2, 3). It affects up to 20% of children and approximately 3% of adults in industrialized countries, making it one of the most common chronic inflammatory skin diseases worldwide (4, 5). A hallmark of AD is the disruption of the epidermal barrier, which results from both structural defects and chronic inflammation. This barrier impairment leads to increased transepidermal water loss, reduced levels of antimicrobial peptides, and greater skin permeability. As a consequence, the skin microbiota loses its normal diversity and balance, creating a permissive environment for the overgrowth of *Staphylococcus aureus* (*S.aureus*) (6, 7). Once dominant, *S. aureus* produces toxins, enzymes, and superantigens that further stimulate immune activation, amplify type 2 inflammation, and ultimately worsen the clinical severity of the disease (8–10). *Staphylococcus aureus* colonization has been consistently associated with increased disease severity, more frequent and persistent relapses, and a poorer response to conventional topical therapies, reflecting its key role in sustaining inflammation and impairing barrier recovery (11, 12). In moderate and severe forms, the response to topical corticosteroids (TCS) and topical immunomodulators may be inadequate, requiring systemic treatments (13). The percentage prevalence of severe AD in the pediatric population is generally less than 5% of all children with AD, with most large international studies reporting rates between 2 and 4.4% depending on the assessment method and geographic region (14). In recent years, monoclonal antibodies targeting type 2 cytokines have revolutionized the therapeutic approach (15). Dupilumab is a fully human monoclonal antibody targeting IL-4Rα, thereby inhibiting both IL-4 and IL-13 signaling and downstream type 2 inflammation. It is currently the only biologic approved for moderate-to-severe AD in children younger than 12 years (16). Dupilumab is approved by both the European Medicines Agency (EMA) and the Italian Medicines Agency (AIFA) for the treatment of severe AD in children starting from 6 months of age (16). Long-term safety data from open-label extension studies, encompassing over 7,000 patient-years of exposure, further support the favorable tolerability of dupilumab in pediatric populations, including infants and young children. The incidence of serious adverse events and infections was not increased with dupilumab compared to placebo, and the safety profile remained stable over up to 5 years of follow-up (17, 18). These findings are reflected in recent reviews and real-world studies, which confirm the efficacy and safety of dupilumab in this age group and support its use as the first approved systemic therapy for severe AD in infants and young children in the EU (19, 20). However, evidence on the impact of dupilumab on the skin and nasal microbiota remains limited, despite the key role of these microbial communities in maintaining epidermal barrier integrity and preventing *S. aureus* colonization and infection (21, 22). The literature indicates that atopic dermatitis is associated with an altered nasal microbiota composition, mainly characterized by enrichment of *S. aureus* and a reduction of commensal staphylococci, with a significant correlation between the nasal microbiome and disease severity (23–25). Nasal carriage of *S. aureus*, particularly when early and persistent during the first months of life, is associated with an increased risk of developing atopic dermatitis and with more severe clinical forms, also correlating with higher total IgE levels and greater extent of skin lesions (24, 26). The nose appears to act as a reservoir for cutaneous recolonization, contributing to the maintenance of Th2-skewed inflammation (23, 24). In contrast, the presence of commensal staphylococci such as *S. epidermidis* and *S. hominis* is associated with a more protective profile, lower systemic inflammation, and clinical improvement, as demonstrated by the increase of these taxa following dupilumab therapy (27). Beyond staphylococci, other nasal taxa, particularly *Moraxella*, also contribute to microbiome compositional differences associated with atopic dermatitis severity in pediatric patients, suggesting that nasal dysbiosis in AD is a complex and multifactorial phenomenon (23). Understanding whether biologic therapy can modulate this microbial ecosystem is therefore crucial. The present study aimed to evaluate the clinical effects of dupilumab in children and adolescents with moderate-to-severe AD through longitudinal assessment of disease severity scores over a 12-month follow-up, and to describe skin and nasal microbial colonization patterns at the 12-month visit. These findings were compared with those observed in a group of patients with moderate AD receiving conventional topical therapy and in healthy controls.

## 2 Methods

### 2.1 Study design and setting

This prospective observational study was conducted at the IPAS Unit (“Respiratory Diseases of Pediatric Interest”), University of Campania “Luigi Vanvitelli,” Naples, Italy. Children and adolescents were consecutively enrolled in the institutional registry between January 2022 and December 2024 and allocated to one of three groups: (a) patients aged 6–16 years with moderate-to-severe atopic dermatitis (AD) for whom dupilumab therapy was initiated; (b) patients in the same age range with moderate AD not eligible for biologic treatment; and (c) healthy age-matched children serving as controls.

The study combined a longitudinal follow-up in dupilumab-treated patients with a cross-sectional comparison at the 12-month time point. Clinical severity scores were analyzed longitudinally only within the dupilumab-treated group, whereas comparisons between groups were performed cross-sectionally at the 12-month visit. Specifically, the dupilumab group underwent standardized clinical evaluations at four predefined visits: T0 (baseline, before treatment initiation), T1 (3 months), T2 (6 months), and T3 (12 months). At T3, nasal and skin samples were collected for culture-based microbial assessment. The moderate AD group and healthy controls underwent a single cross-sectional clinical evaluation and sample collection performed during the same study period in which dupilumab-treated patients reached the 12-month visit (T3), allowing a cross-sectional comparison among the three groups.

Patients in the dupilumab group fulfilled established diagnostic criteria for AD and presented moderate-to-severe disease activity. Dupilumab initiation followed Italian regulatory eligibility criteria for systemic therapy, which include severe disease activity (EASI ≥24) and/or high symptom burden (e.g., Peak Pruritus NRS ≥ 7 or C-DLQI ≥10), as well as involvement of visible or sensitive areas despite optimized topical therapy. Consequently, some patients initiated dupilumab despite baseline EASI values below the severe threshold, reflecting real-world clinical decision-making consistent with national reimbursement criteria. All patients had inadequate disease control with conventional management, including regular use of emollients, topical corticosteroids (TCS), and/or topical calcineurin inhibitors, with persistent symptoms, recurrent flares, and clinically meaningful impairment in quality of life. Dupilumab was administered according to EMA-approved pediatric dosing recommendations based on age and body weight, with an initial loading dose followed by subcutaneous maintenance dosing every 2 or 4 weeks, as appropriate.

Patients in the moderate AD group met diagnostic criteria for atopic dermatitis and had disease severity ranging from EASI 7 to 23. Dupilumab was not initiated either because patients did not meet national reimbursement criteria for biologic therapy or because treatment was declined. They were managed with conventional topical therapy (regular emollients and anti-inflammatory topicals as needed), and disease remained clinically stable with satisfactory symptom control under standard care.

Healthy controls were evaluated at a single time point and included children without personal or family history of AD or other chronic inflammatory, allergic, or immunological conditions, and without current or recent (<6 months) dermatological or systemic diseases. All controls had unremarkable clinical examination findings and were not taking topical or systemic medications at the time of sampling.

The study protocol was approved by the ethics committee of the University of Campania Luigi Vanvitelli (register number 0007134). Written informed consent was obtained from parents or legal guardians before enrollment. Exclusion criteria (all groups) included participation in other clinical studies, presence of chronic systemic diseases or primary immunodeficiencies, use of systemic immunosuppressive drugs or biologics other than dupilumab within the previous 6 months, and presence of active severe or systemic skin infections at the time of enrollment.

### 2.2 Collection of clinical data and questionnaires

At each scheduled study visit (T0, T1, T2, and T3), anthropometric parameters (weight, height, body mass index) and vital signs (blood pressure and heart rate) were systematically recorded. Atopic dermatitis severity was assessed using the Eczema Area and Severity Index (EASI), a validated composite investigator-reported outcome evaluating both the extent and intensity of eczematous lesions. Pruritus intensity was measured using the Peak Pruritus Numerical Rating Scale (NRS; 0–10), a validated patient-reported outcome widely adopted in pediatric AD. Health-related quality of life (QoL) was assessed with the Children’s Dermatology Life Quality Index (C-DLQI), a validated questionnaire exploring the impact of skin disease on daily activities, social interactions, and emotional well-being.

All questionnaires were administered using validated Italian versions. Whenever applicable, children completed the instruments independently; in younger participants, questionnaires were completed with the assistance of a parent or legal guardian to ensure comprehension and reduce missing data.

Microbial colonization assessment was performed at the 12-month visit (T3) through nasal and skin sampling using e-Swab collection systems. Nasal samples were obtained by gently rotating the swab along the anterior nasal mucosa. Skin specimens were collected from active eczematous lesions; in the absence of active lesions, sampling was performed from clinically non-involved skin. In healthy controls, skin samples were collected from standardized anatomical sites corresponding to areas typically involved in atopic dermatitis (e.g., upper limb flexural regions), in order to minimize anatomical variability and improve comparability between groups. Samples were immediately transported under controlled conditions to the Microbiology and Virology Laboratory of the University of Campania “Luigi Vanvitelli” for prompt processing. Use of systemic antibiotics, topical antibiotics, or antiseptic washes within the 4 weeks preceding sampling was an exclusion criterion to minimize potential confounding effects on microbial colonization.

### 2.3 Microbiological procedures

Clinical strains were collected from enrolled patients as reported in paragraph 2.1 and were inoculated in selective and differential culture media. To isolate *S. aureus* and coagulase-negative Staphylococci (including *S. epidermidis*), the isolates were plated on Columbia CNA Agar with 5% Sheep Blood (BioMerieux, Marcy-l’Étoile, France), enabling growth of Gram-positive organisms while suppressing Gram-negative bacteria via colistin and nalidixic acid, and incubated overnight at 37 °C. Bacterial identification and susceptibility tests were worked out using Matrix-Assisted Laser Desorption/Ionization–Time of Flight (MALDI-TOF, Bruker Daltonics, Bremen, Germany) and the Phoenix BD system (Becton Dickinson, Franklin Lakes, NJ, USA). After 16 h of incubation, the resistance patterns were interpreted according to the EUCAST breakpoints.

### 2.4 Statistical analysis

Continuous variables were assessed for normality using the Shapiro–Wilk test. As data were not normally distributed, longitudinal changes in EASI, C-DLQI, and Peak Pruritus NRS scores within the dupilumab-treated group were analyzed using the Friedman test for repeated measures. Continuous variables are presented as median (IQR; minimum–maximum), while categorical variables are reported as counts and percentages. Statistical significance was set at a two-sided *p* value <0.05. Data were analyzed using Microsoft Excel (Microsoft 365, Microsoft Corporation, Redmond, WA, USA) and GraphPad Prism (version 8.0.2; GraphPad Software, San Diego, CA, USA).

## 3 Results

### 3.1 Study population

A total of 30 participants were included in the analysis: 10 children with moderate-to-severe AD treated with dupilumab, 10 age-matched children with moderate AD receiving conventional topical therapy, and 10 healthy controls. The median age in the moderate-to-severe AD treated with dupilumab group was 13 years; sex distribution was comparable across groups. No participant was lost to follow-up.

### 3.2 Clinical outcomes in dupilumab-treated patients

#### 3.2.1 Disease severity (EASI)

**Figure 1:**
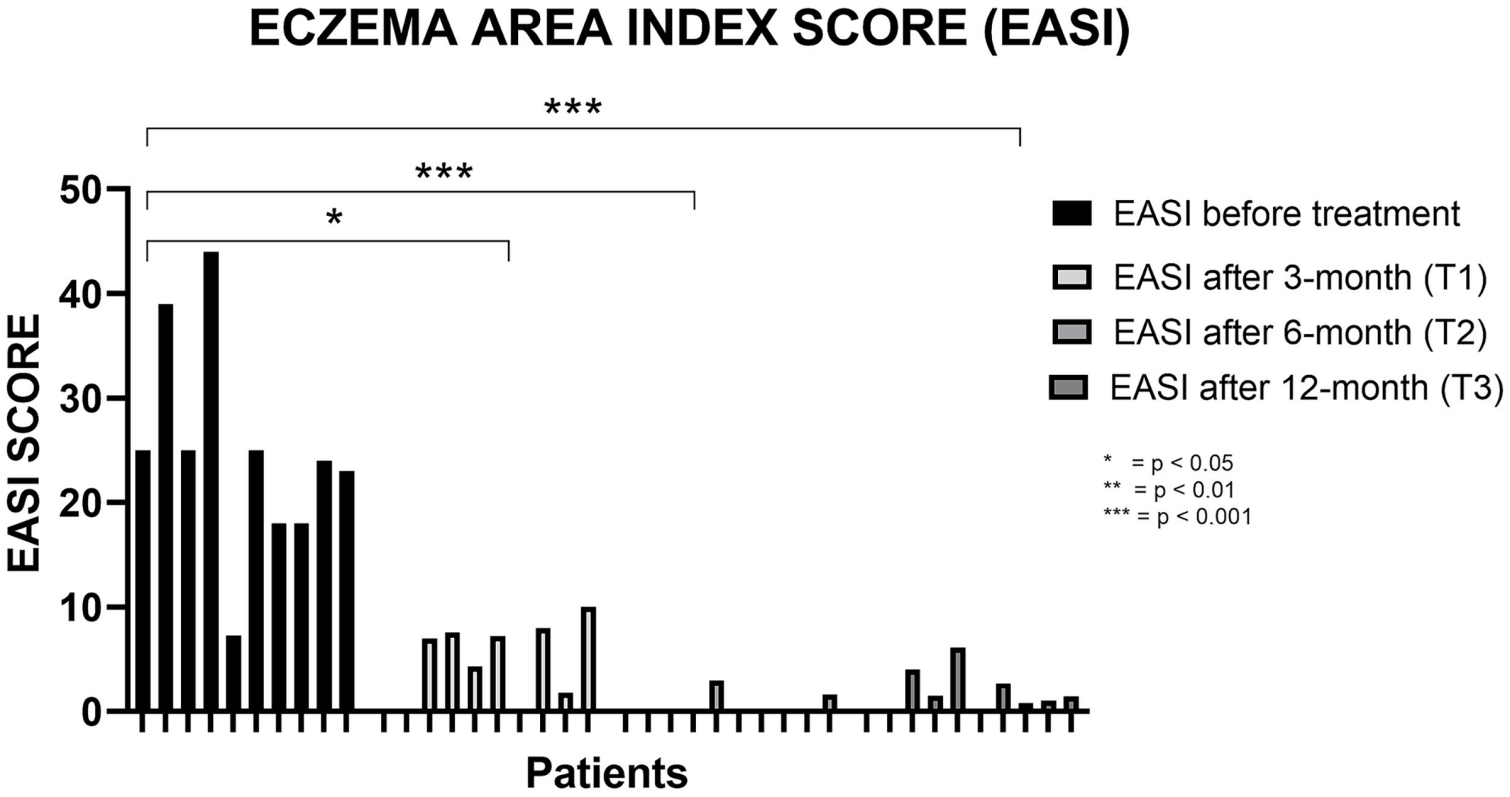
Trend of the EASI score before treatment and after 3, 6, and 12 months of dupilumab. EASI, Eczema Area Severity Index. The asterisks indicate the level of statistical significance: *p < 0.05; **p < 0.01; ***p < 0.001.

Dupilumab treatment was associated with a rapid and sustained improvement in AD severity over 12 months. The median EASI decreased from 24.5 (IQR 18.0–28.5; range 7.3–44.0) at baseline to 5.65 (IQR 0.0–7.7; range 0.0–10.0) at 3 months, reaching 0.0 (IQR 0.0–0.4; range 0.0–3.0) at 6 months and remaining low at 12 months (median 1.2; IQR 0.0–3.03; range 0.0–6.1) (Figure 1). Overall longitudinal changes were statistically significant (Friedman test, *p* < 0.05) (Table 1).

**Table 1:** Clinical outcomes and culture-based *S. aureus* colonization.

Outcome/site	Baseline (T0)	3 months (T1)	6 months (T2)	12 months (T3)
EASI	24.5 (18.0–28.5; 7.3–44.0)	5.65 (0.0–7.7; 0.0–10.0)	0.0 (0.0–0.4; 0.0–3.0)	1.2 (0.0–3.03; 0.0–6.1)
C-DLQI	13.5 (9.25–16.5; 5–23)	3.0 (0.75–7.25; 0–13)	2.0 (0.75–5.5; 0–10)	3.5 (0.0–11.25; 0–14)
Peak pruritus NRS	10.0 (8.75–10.0; 8–10)	4.5 (0.75–7.0; 0–7)	3.0 (0.75–5.0; 0–7)	5.5 (0.0–7.25; 0–8)

Culture-based *S. aureus* colonization at 12 months (T3)			
Site	Dupilumab-treated AD (*n* = 10)	Moderate AD receiving conventional topical therapy (*n* = 10)	Healthy controls (*n* = 10)
Skin swab: *S. aureus* positive, *n* (%)	0 (0%)	8 (80%)	0 (0%)
Nasal swab: *S. aureus* positive, *n* (%)	2 (20%)	4 (40%)	1 (10%)

#### 3.2.2 Quality of life (C-DLQI)

**Figure 2:**
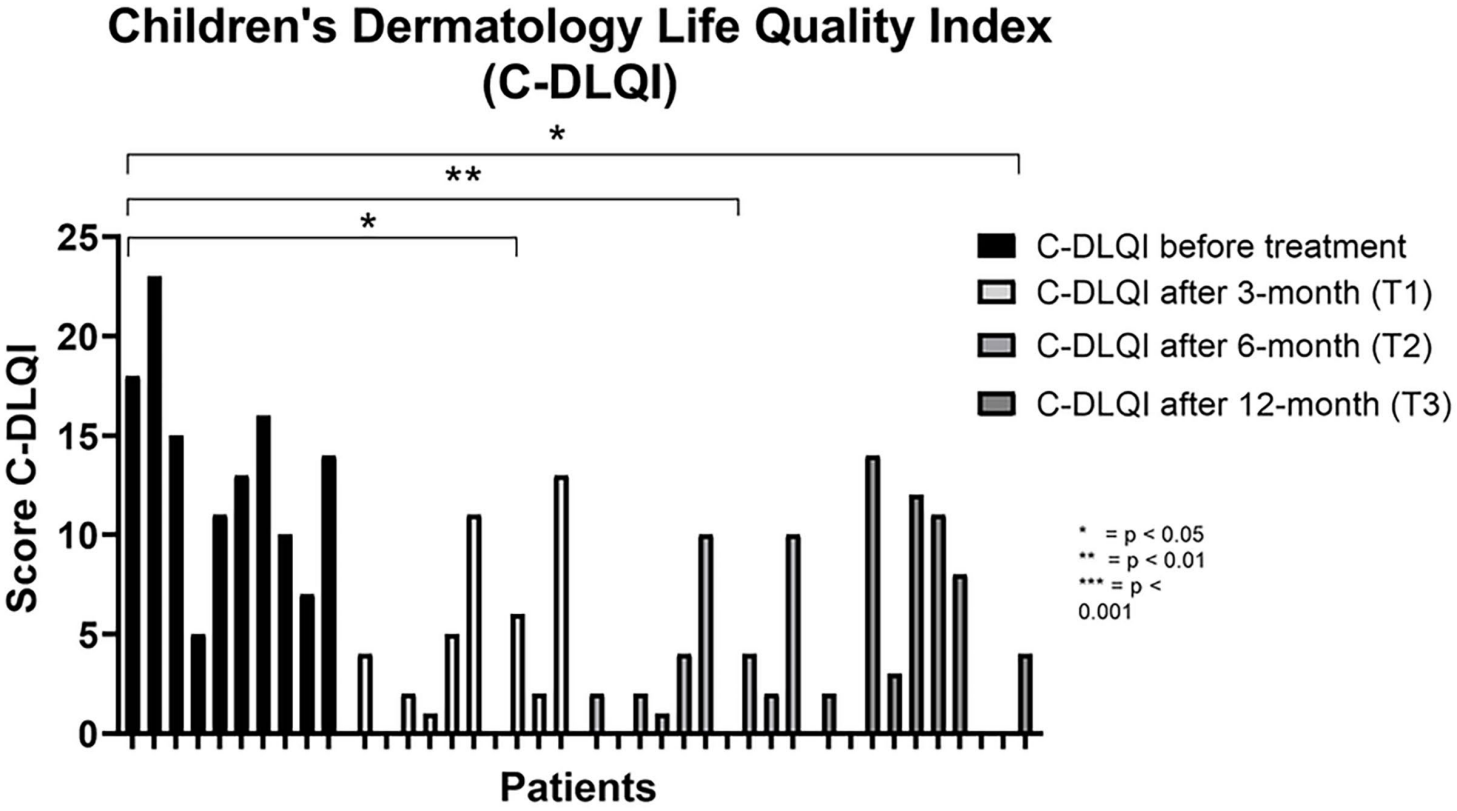
Trend of the C-DLQI score before treatment and after 3, 6, and 12 months of dupilumab. C-DLQI, Children’s Dermatology Life Quality Index. The asterisks indicate the level of statistical significance: *p < 0.05; **p < 0.01; ***p < 0.001.

Health-related quality of life markedly improved from the earliest follow-up visits. Median C-DLQI declined from 13.5 (IQR 9.25–16.5; range 5–23) at baseline to 3.0 (IQR 0.75–7.25; range 0–13) at 3 months and to 2.0 (IQR 0.75–5.5; range 0–10) at 6 months. At 12 months, C-DLQI remained substantially improved compared with baseline (median 3.5; IQR 0.0–11.25; range 0–14) (Figure 2) (Friedman test, *p* < 0.05) (Table 1).

#### 3.2.3 Pruritus intensity (peak pruritus NRS)

**Figure 3:**
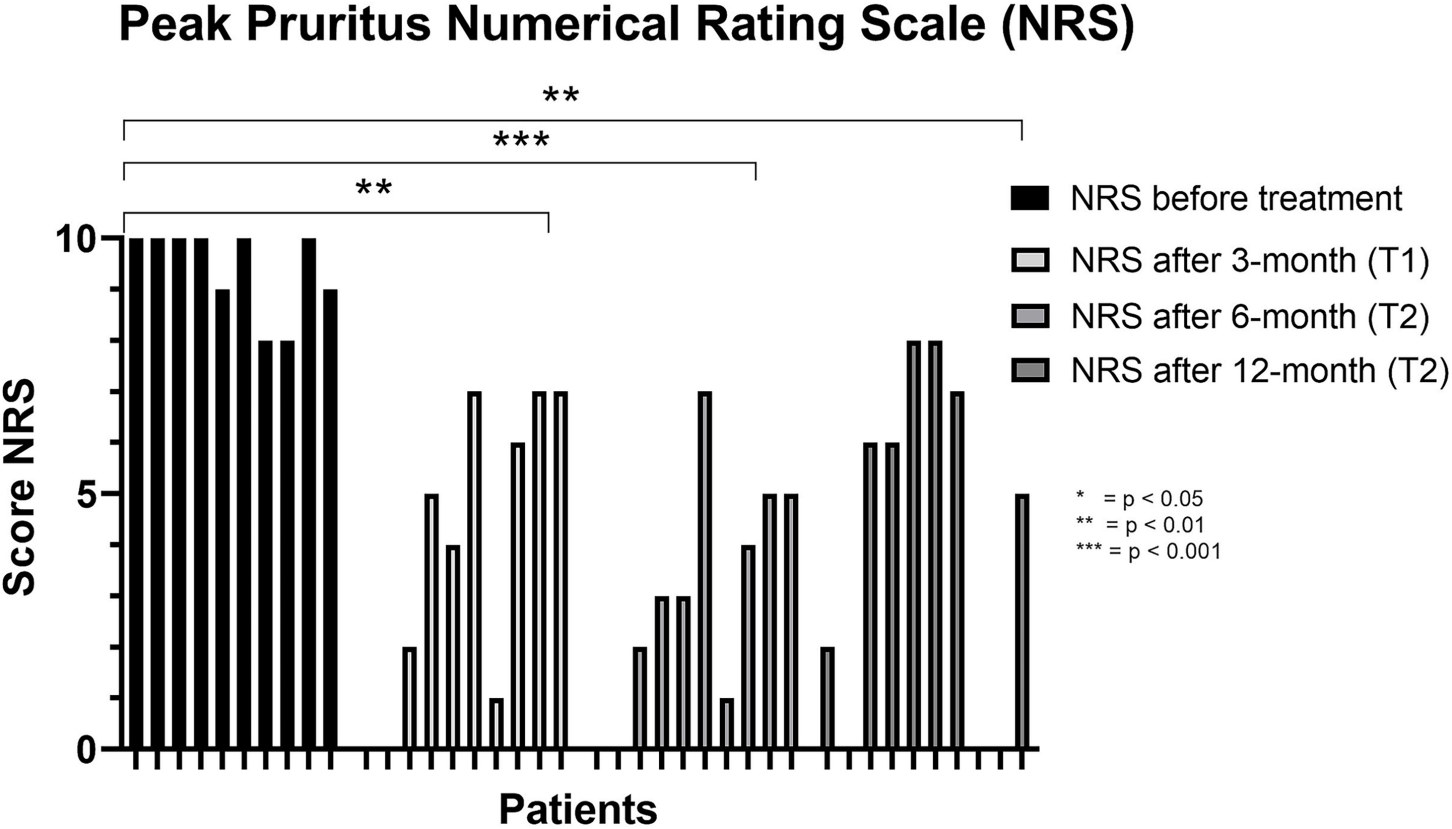
Trend of the NRS score before treatment and after 3, 6, and 12 months of dupilumab. NRS, Peak Pruritus Numerical Rating Scale.

Pruritus severity also decreased substantially during treatment. Median Peak Pruritus NRS improved from 10.0 (IQR 8.75–10.0; range 8–10) at baseline to 4.5 (IQR 0.75–7.0; range 0–7) at 3 months and 3.0 (IQR 0.75–5.0; range 0–7) at 6 months. At 12 months, a modest increase was observed (median 5.5; IQR 0.0–7.25; range 0–8), although scores remained lower than baseline (Friedman test, *p* < 0.05) (Figure 3 and Table 1). No dupilumab-related adverse events were recorded, and no patient discontinued therapy during follow-up.

### 3.3 Culture-based assessment of nasal and skin colonization

Microbial colonization was evaluated at the 12-month visit (T3) using culture-based methods (Table 1).

#### 3.3.1 Skin swabs

**Figure 4:**
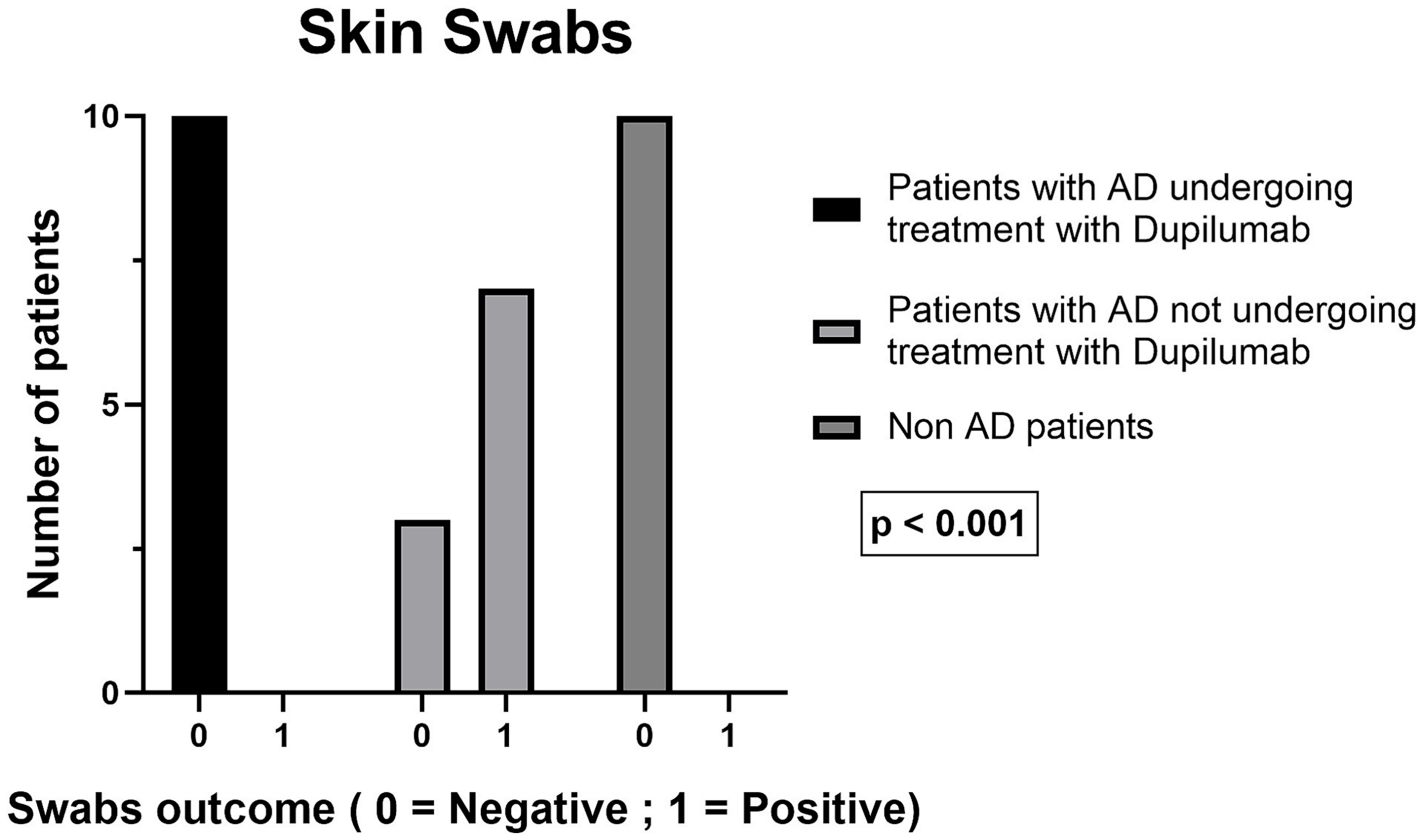
Number of patients with skin swabs positive for *Staphylococcus aureus* in the three groups (dupilumab, moderate AD receiving conventional topical therapy, controls). The asterisks indicate the level of statistical significance: *p < 0.05; **p < 0.01; ***p < 0.001.

Skin *S. aureus* colonization was not detected in moderate-to-severe AD treated with dupilumab patients (0/10), whereas it was present in 8/10 children in the moderate AD receiving conventional topical therapy group. No skin *S. aureus* colonization was observed among healthy controls (0/10). In dupilumab-treated patients, the absence of *S. aureus* was accompanied by the presence of commensal bacteria (including *Staphylococcus epidermidis*, *Cutibacterium* spp., and *Micrococcus luteus*), based on qualitative culture-based identification (Figure 4).

#### 3.3.2 Nasal swabs

**Figure 5:**
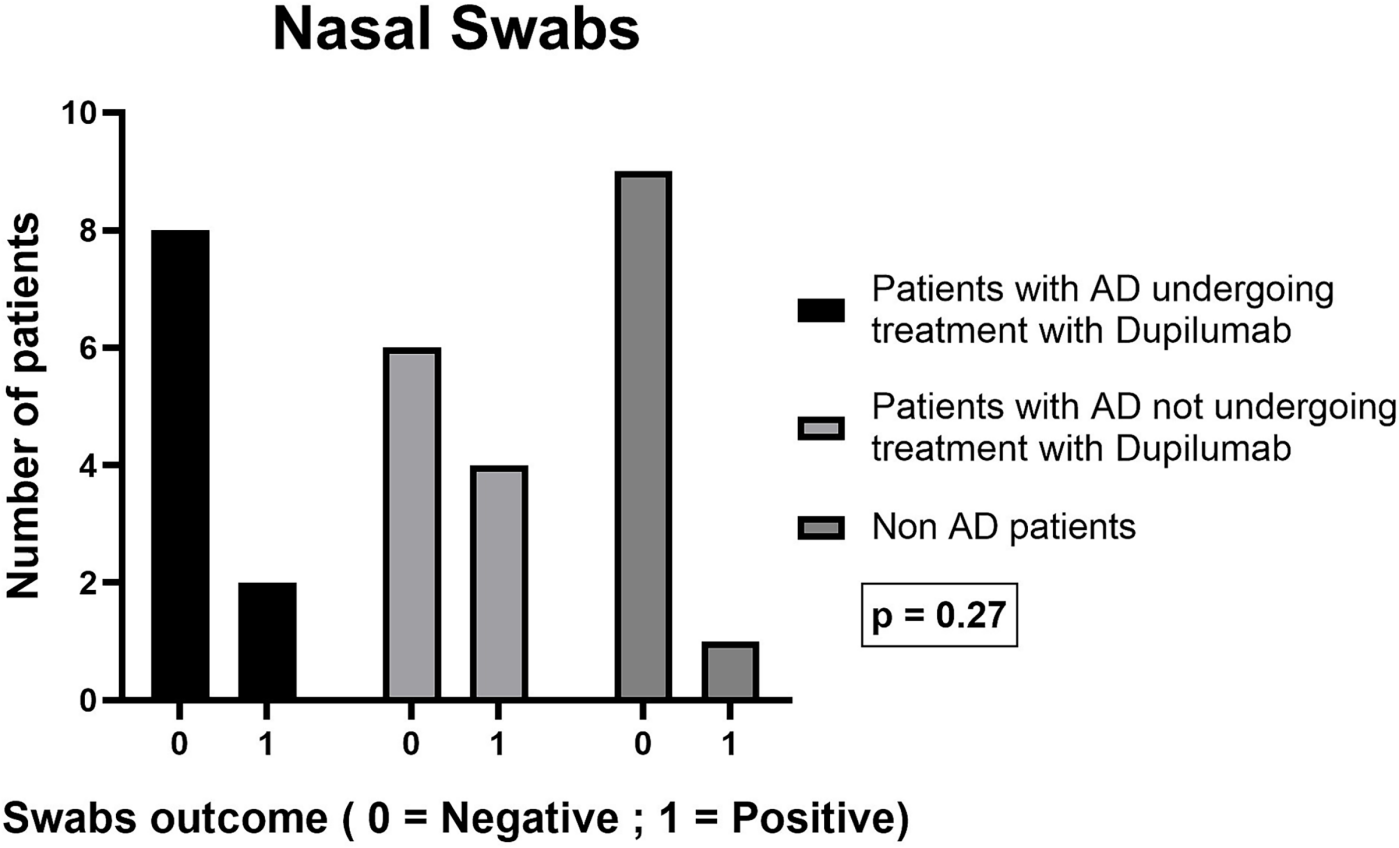
Number of patients with nasal swabs positive for *Staphylococcus aureus* in the three groups (dupilumab, moderate AD receiving conventional topical therapy, controls).

Nasal *S. aureus* colonization was detected in 2/10 dupilumab-treated patients, compared with 4/10 in the moderate AD receiving conventional topical therapy group and 1/10 among healthy controls (*p* = 0.27) (Figure 5).

Overall, compared with moderate AD receiving conventional topical therapy, moderate-to-severe AD treated with dupilumab patients showed a lower frequency of *S. aureus* colonization at both nasal and skin sites, consistent with a pattern closer to that observed in healthy controls.

## 4 Discussion

This prospective observational study evaluated the effectiveness of 12 months of dupilumab treatment in children with moderate-to-severe atopic dermatitis and explored, in our clinical setting, its association with changes in culture-based nasal and skin microbial colonization profiles. Overall, dupilumab induced a rapid and marked improvement in disease severity, pruritus, and quality of life, with the greatest reduction in EASI, C-DLQI, and NRS scores occurring within the first 6 months, in line with evidence from pediatric trials and real-world studies showing early, clinically meaningful benefit under IL-4Rα blockade (28, 29). Scores remained low at 12 months; however, a slight increase in NRS and C-DLQI was observed at the final follow-up. This finding may reflect multiple factors, including variability in disease course, seasonal influences, or changes in adherence to supportive topical therapy, and should be interpreted with caution. In routine practice, perceived well-being may lead some patients and caregivers to prematurely decrease regular emollient and topical maintenance therapy, potentially contributing to mild symptom recurrence. This observation reinforces the importance of maintaining barrier-supportive treatment alongside biologic therapy. Beyond clinical response, a key finding of our study is the lower prevalence of *S. aureus* colonization in dupilumab-treated patients compared with children managed with conventional topical therapy. Given the cross-sectional nature of the microbiological assessment at the 12-month time point, these findings should be interpreted with appropriate caution. Notably, the absence of *S. aureus* in skin swabs from dupilumab-treated patients was not associated with a decrease in overall bacterial growth, but rather with the presence of commensal taxa, including *S. epidermidis*, *Cutibacterium* spp., and *M. luteus*. Importantly, commensal microbiota findings in this study are limited to qualitative (presence/absence) culture-based identification and do not allow conclusions regarding relative abundance. These observations suggest that, in our cohort, lower *S. aureus* colonization was observed alongside the presence of a physiologic cutaneous colonization profile near lesional skin. These findings are consistent with previous studies suggesting that IL-4/IL-13 pathway inhibition may be associated with reduced *S. aureus* colonization and a shift toward a more balanced microbial colonization profile (30–32). Across studies in adults with moderate-to-severe AD, IL-4/IL-13 pathway inhibition has been associated with rapid normalization of cutaneous microbial imbalance, characterized by decreased *S. aureus*, increased microbial diversity, and improvements in clinical outcomes (30–32). In a randomized, placebo-controlled trial, dupilumab induced a significant reduction in *S. aureus* load as early as day 3, preceding overt clinical improvement, and reductions in *S. aureus* were associated with better clinical outcomes and correlated with decreases in type 2 inflammatory biomarkers such as CCL17/TARC, together with reductions in staphylococcal cytotoxins and inflammatory transcriptomic signatures (31). Similarly, another randomized trial reported increased microbial diversity and reduced *S. aureus* abundance (including qPCR-based measurements) during dupilumab therapy, with significant correlations between staphylococcal abundance, disease severity indices (EASI/SCORAD), and type 2 biomarkers (particularly TARC/CCL17 and PARC/CCL18); notably, treatment discontinuation was accompanied by a tendency toward reversion to pre-treatment microbiological patterns (30). Finally, in a prospective cohort of adults with moderate-to-severe AD, clinical improvement under dupilumab was accompanied by strengthening of barrier parameters (including reduced lesional TEWL and increased stratum corneum hydration), as well as changes in lipid composition and antimicrobial peptide expression; in parallel, decreased *S. aureus* and increased commensal taxa were observed, supporting a link between barrier recovery and microbial remodeling (32).

In addition to these findings, integrated sequencing-based studies have further strengthened the evidence for microbiome remodeling under dupilumab. In a prospective cohort of 30 treated patients followed for 12 weeks, combined bacteriome and mycobiome profiling documented an increase in microbial diversity during therapy, with a reduction in *S. aureus* colonization and broader shifts in fungal communities, including changes in Malassezia abundance correlating with disease severity and systemic type 2 biomarkers such as serum TARC (33). On a larger scale, real-world data from the TREATgermany registry using 16S sequencing before and after 3 months of systemic therapy showed that dupilumab but not cyclosporine shifted the bacterial community toward a healthy-control pattern, reducing staphylococci*/S. aureus* and increasing potentially protective commensals such as *Staphylococcus hominis* (34). Notably, these microbial changes appeared partially independent of individual clinical improvement, suggesting that IL-4Rα blockade may directly influence the cutaneous microenvironment and host–microbiota interactions (34).

Taken together, these data support the concept that inhibition of IL-4/IL-13 signaling through IL-4Rα blockade may contribute to a more balanced cutaneous microbial profile, reducing potentially pathogenic taxa and favoring commensals. This biological plausibility is further supported by evidence that IL-4 and IL-13 suppress antimicrobial peptide production and disrupt keratinocyte cohesion, thereby facilitating *S. aureus* overgrowth; conversely, cytokine blockade may improve host defenses and barrier function, promoting commensal recolonization (35). In this context, our culture-based findings in children, demonstrating lower levels of skin *S. aureus* colonization compared with patients with moderate AD managed with conventional topical therapy, are consistent with mechanisms proposed by sequencing-based studies in adults, while preserving a clinically relevant and pragmatic approach to microbial assessment.

In contrast to the substantial cutaneous signal, available evidence on mucosal sites suggests more variable effects. Although several AD studies indicate reductions in nasal *S. aureus* carriage, microbiome-level shifts in low-biomass compartments may be less pronounced and more difficult to detect. In a longitudinal pilot study of patients with N-ERD assessed by 16S sequencing, despite clinical improvement under dupilumab, no consistent changes in alpha-diversity or overall nasal composition were detected, and a reduction in staphylococci was observed only in a minority of subjects; the authors emphasized the technical challenges intrinsic to low-biomass sample analysis (36). Although N-ERD differs from AD and direct extrapolation is not possible, these data support cautious interpretation of nasal results and highlight the importance of methodological rigor when investigating mucosal microbial communities. Pediatric evidence remains limited. Interestingly, a pediatric case report in a child with genetically confirmed Netherton syndrome treated off-label with dupilumab documented marked clinical improvement alongside an increase in skin microbial alpha-diversity and a reduction in *S. aureus* abundance on affected skin, suggesting that IL-4/IL-13 pathway modulation may facilitate a favorable microbial shift even in severe barrier disorders (37). While such evidence is inherently limited, it provides additional biological plausibility supporting the observed association between clinical improvement and microbial rebalancing. Emerging literature also suggests that dupilumab may modulate microbial ecosystems beyond the skin, potentially involving a broader skin–mucosa axis. In an observational cohort, 16S rRNA sequencing demonstrated gut dysbiosis in AD compared with controls and showed that dupilumab therapy was associated with a shift toward a more “healthy-like” intestinal profile, with changes in microbial network connectivity and increases in potentially beneficial genera correlating with severity indices and inflammatory biomarkers such as TARC (38). Functional analyses suggested modulation of tryptophan metabolism and increased levels of indole pathway metabolites, providing a mechanistic rationale for interactions between gut microbiota, immune modulation, and systemic inflammation (38). While exploratory and not directly assessed in our study, these findings raise the hypothesis that IL-4Rα blockade may influence host–microbiota interactions at multiple sites (38).

### 4.1 Limitations and future research directions

This study has several strengths. The inclusion of three well-defined groups (dupilumab-treated moderate-to-severe AD, moderate AD receiving conventional topical therapy, and healthy controls) allows direct comparison of clinical and microbial outcomes across different disease severities and treatment exposures. The 12-month longitudinal follow-up of treated patients provides insight into sustained clinical response, while the cross-sectional comparison at 12 months provides pragmatic evidence of microbial colonization patterns under stable treatment conditions. Nevertheless, limitations must be considered. The small sample size represents an important limitation of this study and reduces the statistical power of the analyses. In particular, the limited cohort size may have prevented potentially meaningful differences in microbial colonization patterns from reaching statistical significance. Importantly, microbial sampling was performed only at 12 months, preventing baseline comparison and limiting the characterization of early dynamics. Finally, microbial assessment was culture-based and therefore does not capture overall microbiome diversity, functional pathways, or non-cultivable taxa. In addition, the study evaluated microbial colonization without systematically assessing clinically documented secondary skin infections or direct correlations between colonization patterns and disease exacerbations. Therefore, the clinical implications of the observed microbiological findings should be interpreted with caution, as microbial assessment was performed at a single time point (12 months) without baseline measurements. Consequently, it cannot be definitively established whether the observed colonization patterns were pre-existing or related to treatment. Larger pediatric studies with longitudinal sampling at multiple time points and integrative sequencing-based approaches are warranted to clarify the temporal relationship between clinical response and microbial remodeling and to explore the long-term implications of microbial changes during biologic therapy.

## 5 Conclusion

Dupilumab appears to be a safe and effective treatment for pediatric moderate-to-severe atopic dermatitis. In this study, dupilumab treatment was associated with rapid improvement in pruritus, disease severity, and quality of life as early as 3 months, with sustained benefit at 12 months. Culture-based assessment also showed lower levels of *S. aureus* colonization at both skin and nasal sites, along with the presence of commensal staphylococci based on culture-based identification. These findings support the hypothesis that dupilumab may contribute not only to controlling type 2 inflammation but also to restoring a more physiologic microbial balance associated with skin barrier health. Although limited by the small sample size and the single-center, non-randomized design, our real-world experience suggests that dupilumab is a valuable therapeutic option for children with moderate-to-severe AD. Maintaining appropriate baseline skincare measures, including regular emollient use, remains essential to optimize long-term outcomes. Larger prospective studies are warranted to confirm these observations and to investigate the durability of microbial changes and their long-term clinical implications.

## Data Availability

The original contributions presented in the study are included in the article/supplementary material, further inquiries can be directed to the corresponding author.
